# Comparison of Hyaluronate & Steroid Injection in the Treatment of Chronic Lateral Epicondylitis and Evaluation of Treatment Efficacy With MRI: A Single-Blind, Prospective, Randomized Controlled Clinical Study

**DOI:** 10.7759/cureus.29011

**Published:** 2022-09-10

**Authors:** Atilla Yalcin, M. Enes Kayaalp

**Affiliations:** 1 Orthopaedics and Traumatology, Istanbul Taksim Research and Training Hospital, Istanbul, TUR; 2 Orthopaedics and Traumatology, Istanbul Kartal Research and Training Hospital, Istanbul, TUR

**Keywords:** hyaluronic acid, steroids, triamcinolone, prospective studies, tennis elbow

## Abstract

Introduction: Injection therapy in refractory cases of lateral epicondylitis might relieve symptoms, although no consensus exists on which material to use. Corticosteroids are widely used but recent literature indicated possible tenotoxic effects and inefficacy in mid- and long-term follow-up (FU). Hyaluronate/hyaluronic acid (HA) might be of better clinical efficacy. Magnetic resonance imaging (MRI) might reflect the clinical changes in the short-term FU.

Methods: A single-blind, prospective, randomized controlled study was designed. A total of 80 patients were included. A total of 40 patients received a single triamcinolone injection and 40 received a hyaluronic acid (HA) injection. Follow-up was repeated at six and 12 weeks. The shortened disabilities of the arm, shoulder, and hand questionnaire (Q-DASH) score; visual analog scale (VAS) for pain at rest, and hand grip strength were collected. Dynamometer measurements were done at baseline and FU examinations. The MRI images at baseline and 12 weeks FU were evaluated.

Results: There were significant differences between the groups favoring the triamcinolone group at six weeks. At 12 weeks, no differences existed between the groups in any of the parameters. The MRI grades were nonsignificantly different between baseline and at 12 weeks.

Conclusion: Both triamcinolone and HA were shown to relieve pain and increase functional outcomes. However, the effects were short-lived. The MRI did not show significant differences at 12 weeks, although clinical improvements were substantial.

## Introduction

Lateral epicondylitis (LE), also known as tennis elbow is one of the most common musculoskeletal conditions in daily practice and the most common cause of lateral elbow pain affecting mainly middle-aged people and up to 3% of the population [1-3]. The pathophysiology of the condition is multifactorial and subject to much debate with overuse constituting a common etiological factor [4]. There is no consensus on which conservative therapy measure to use as the first-line therapy, as wait-and-see protocols were also shown to be efficient in significant symptom resolution [1,2]. 

On the other hand, injection therapies using corticosteroids (CS), platelet-rich plasma (PRP), and autologous blood have been widely reported in the literature with conflicting clinical efficacy [2,5]. Among injected materials, CS are commonly reported to relieve pain and increase functional outcomes in the short term with no or less prominent efficacy and potentially harmful effects in the long term [6,7]. However, no consensus exists with regard to the optimal injection material to treat LE [8]. 

Recently, growing evidence indicated that CS can have tenotoxic effects, increasing the risk of tendon or ligament rupture while increasing necrosis and decreasing cell viability [9-11]. These concerns lead to the use of other materials for injection therapies. One of these materials is hyaluronic acid (HA) or hyaluronate. Hyaluronic acid is widely used in orthopedic practice, although its efficacy is questionable [12]. However, HA injections are known for their relatively safe profile and have been shown to treat tendinopathies [13]. On the other hand, high costs and concerns about their efficacy make their adoption less likely. Currently, more studies are needed to draw conclusions on their efficacy in LE [6]. 

The LE diagnosis is mostly based on subjective clinical examination [14]. A recent network meta-analysis underlined the importance of valid diagnostic inclusion criteria in clinical trials regarding LE, which should include confirmation of diagnosis with imaging [8]. Magnetic resonance imaging (MRI) constitutes an objective diagnostic method in LE [15]. An MRI has also been shown to reflect a response to treatment over the course of treatment [16]. Therefore the current study incorporated confirming MRI findings as inclusion criteria and a method of diagnostic follow-up.

In summary, there is currently a lack of clinical trials with a high level of evidence on which to base recommendations regarding injection therapy in chronic LE [8]. Therefore, this study aimed to: (1) compare the clinical efficacy of a single injection with triamcinolone or hyaluronate in LE, and (2) evaluate the treatment effect using MRI in the short term by incorporating a randomized, single-blind study protocol.

The hypothesis was that hyaluronic acid would have better alleviation of symptoms and would be associated with better functional outcomes with no adverse effects as would be apparent on follow-up MRI.

## Materials and methods

**Figure 1:**
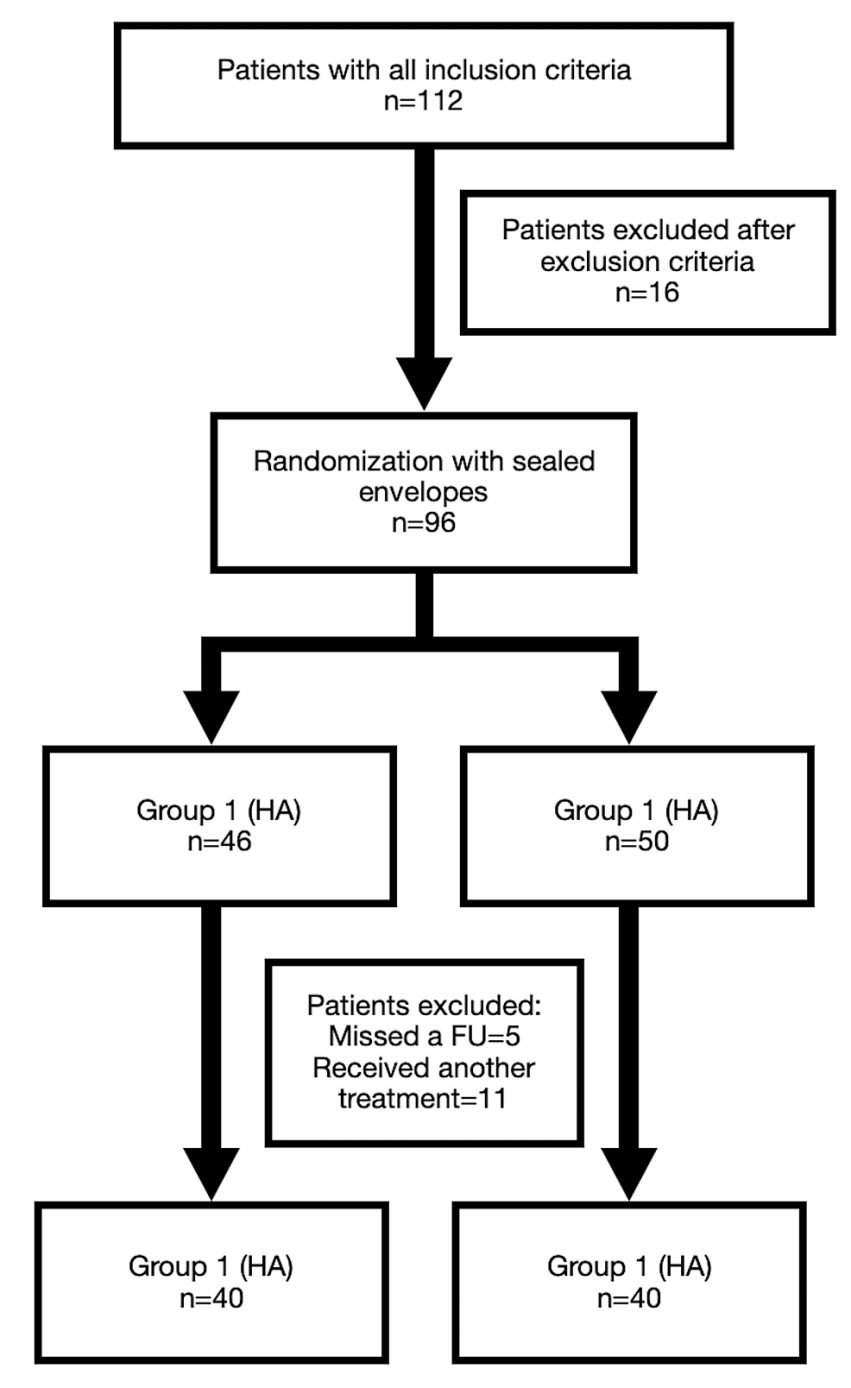
The flowchart of the included patients HA: Hyaluronate, FU: Follow-up

An independent Institutional Ethical Committee's approval was obtained for this prospective randomized study (No:254, Date:09.06.2021). The flowchart of patients is represented in Figure 1. A total of 40 patients in each group with all FU examinations were included in the data analysis. All patients had chronic lateral epicondylitis with a symptom duration of at least three months and positive MRI findings. Patient demographics are represented in Table 1.

**Table 1:** Patient demographics CS: Corticosteroid, HA: Hyaluronate, *: Independent sample t-test, **: Chi-square

		CS	HA	p-value
Age		36.42±12.11	34.35±10.89	0.563*
Gender				
	Female	21	20	0.823**
	Male	19	20	
Affected side				
	Left	11	9	0.605**
	Right	29	31	
Dominant side				
	Left	6	5	0.745**
	Right	34	35	

The clinical diagnosis was made with the presence of at least two of the three clinical evaluation measures, Cozen’s, Mill’s, and Maudley’s tests. Inclusion criteria for patients were: (1) aged 20 to 50 years old, (2) clinical diagnosis of LE with at least two positive clinical tests, (3) supportive MRI findings of LE, (4) visual analog scale (VAS) for pain at hand grip strength of at least 5 out of 10 at the initial diagnosis. Exclusion criteria were: (1) prior injection/acupuncture or recent physical therapy to the elbow, (2) inflammatory or rheumatological disease diagnosis, (3) nerve entrapment or cervical radiculopathy, (4) allergies to CS or birds, or egg products, (5) missing FU examination.

**Figure 2:**
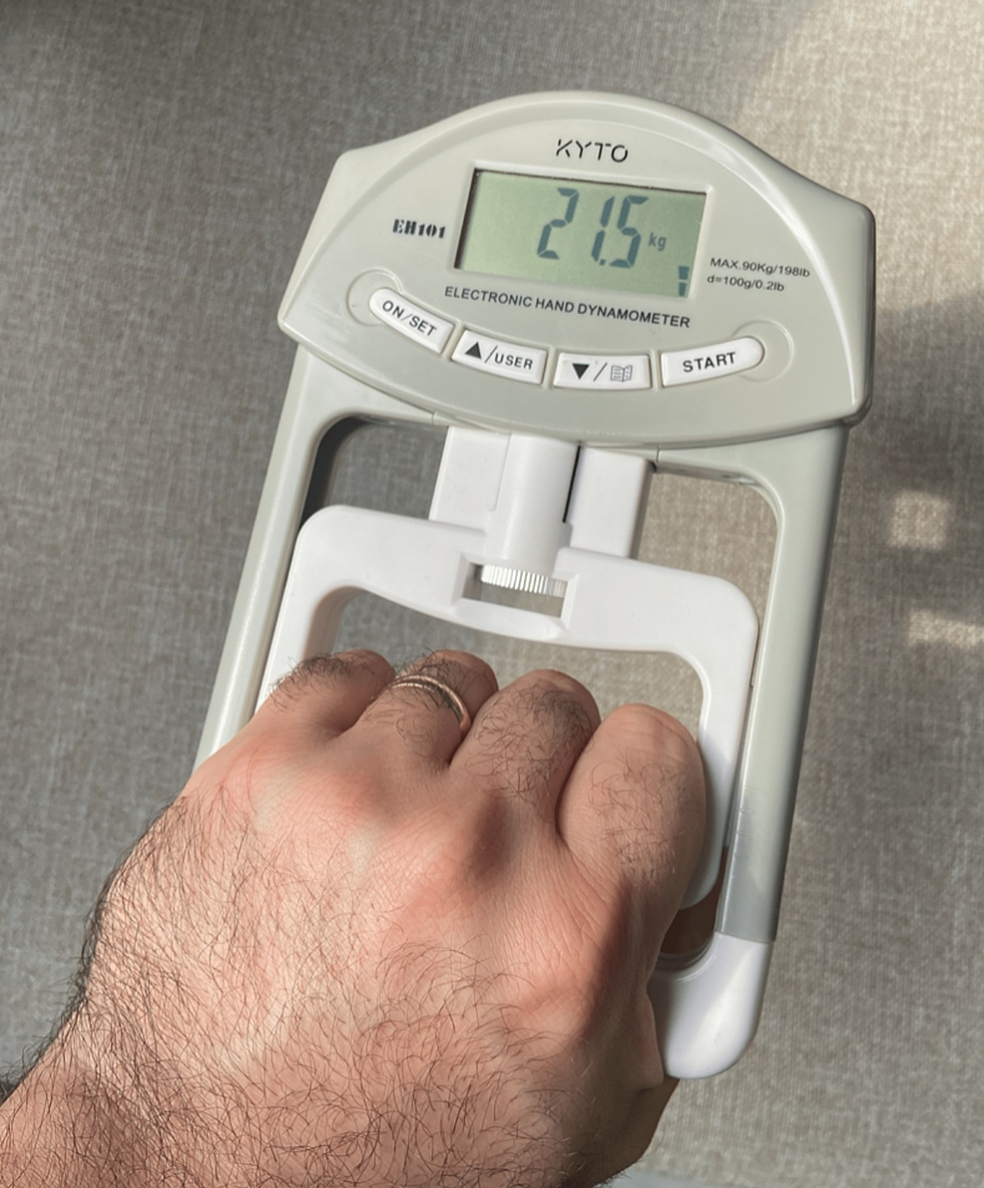
A digital hand dynamometer was used to measure hand grip strength

The patients were randomized into one of the two groups using simple randomization in a 1:1 fashion and using sealed envelopes. Patients received either a single HA injection (30mg/2mL 2000kDa high-molecular-weight) or 5 mg triamcinolone (5mg/2mL) at the most tender location of their lateral elbow. All injections were performed by the first author, who was blinded to injection material. All patients were followed-up until 12 weeks. Primary outcome measures were the shortened disabilities of the arm, shoulder, and hand questionnaire (Q-DASH) score and VAS for pain at rest and activity. These scores were collected and hand grip strength was measured using an electronic hand dynamometer (Figure 2) at the following time points: prior to injection (baseline), at six weeks, and 12 weeks after the injection. Every time before use, the electronic dynamometer was restarted for calibration. The elbow position was relaxed extension during dynamometer measurements and they were repeated three times at each time point [1]. Mean values were used for data analysis. A follow-up MRI was ordered for the 12-week follow-up. A standardized MRI grading of tendinopathy was completed as suggested in previous studies [16,17]. Images were graded depending on the tendon signals on MRI. The grading was completed both at baseline and 12 weeks by a radiologist experienced in musculoskeletal diseases. The MRI images were graded as normal (0), mild (1), moderate (2), or severe (3) [16,17]. No complimentary medications in any form nor physical therapy were allowed during the study. 

Demographic data were compared using an independent sample t-test and Chi-square test. Baseline scores were compared using Student’s t-test. Differences in repeated measures were compared using paired sample t-test. A posthoc power analysis using G*Power 3.1 software (Düsseldorf, Germany) incorporating the mean Q-DASH scores in follow-up showed alpha=0.05 and beta=0.99 for 40 patients in each group.

## Results

All patients were followed-up until 12 weeks. There were no differences in any evaluated parameters at the baseline examination (Table 2). There were significant differences at the six-week FU and nonsignificant differences at the 12-week FU in evaluated parameters (Table 2). 

**Table 2:** Outcome parameters at baseline and follow-up examinations Δ: Difference, HA: Hyaluronate, CS: Corticosteroid, SD: Standard deviation, n.a.: not applicable, *: Student's t-test, Q-DASH: The shortened disabilities of the arm, shoulder, and hand questionnaire

	CS	HA	Δ (HA versus CS)	p-value*
Outcome and time points	Mean±SD	Mean±SD	Mean improvement from baseline±SD	Difference between groups
Pain at rest				
Baseline	6.39±0.8	6.34±0.73	n.a.	0.774
Week 6	2.85±0.91	3.37±0.99	0.56±2.09	0.017
Week 12	4.07±1.08	3.88±1	-0.14±1.53	0.400
Pain with hand grip				
Baseline	7.54±0.99	7.2±0.81	n.a.	0.750
Week 6	3.49±1	4.07±0.93	0.93±1.95	0.008
Week 12	4.41±0.97	4.22±1.1	0.56±2.09	0.377
Q-DASH				
Baseline	59.27±9.03	54.61±8.11	n.a.	0.676
Week 6	33.02±7.4	41.22±8.06	12.85±19.83	0.001
Week 12	43.22±10.34	38.36±7.39	-0.19±10.71	0.102
Grip strength				
Baseline	21.25±3.43	19.95±4.46	n.a.	0.142
Week 6	40.44±2.56	38.88±2.18	-0.26±4.6	0.004
Week 12	38.82±3	37.1±2.54	-0.05±8.33	0.345

**Figure 3:**
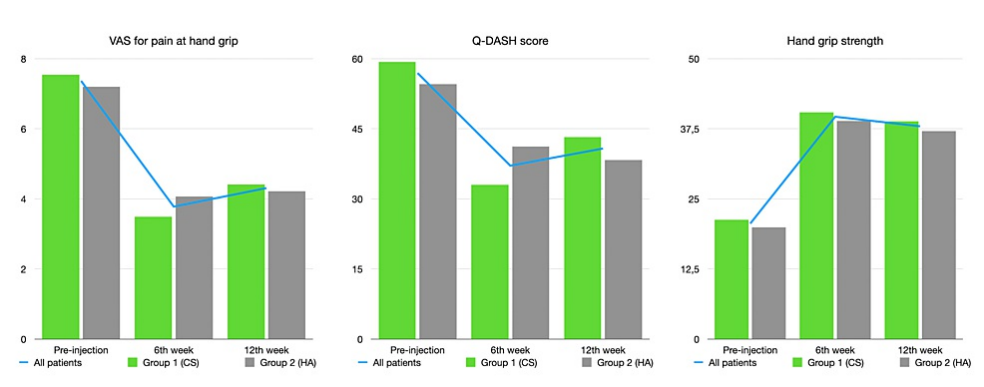
Graphical changes in outcome measures from left to right: VAS for pain at hand grip, Q-DASH, and hand grip strength Q-DASH: The shortened disabilities of the arm, shoulder, and hand questionnaire, VAS: Visual analog scale, CS: Corticosteroid, HA: Hyaluronate

The changes in evaluated parameters throughout the study and between different time points are represented in Table 3 and shown in Figure 3. There were significant differences between both groups regarding changes in parameters at different time points. Changes were more prominent in the CS group for the first six weeks of FU followed by a slower worsening in evaluated parameters until 12 weeks. The effect patterns of CS and HA were significantly different as was apparent from p-values for changes in outcome parameters between follow-up time points.

**Table 3:** The changes in outcome measures between evaluation time points CS: Corticosteroid, HA: Hyaluronate, SD: Standard deviation, Δ: Change in the evaluated parameter in the corresponding unit, *: Paired sample t-test, Q-DASH: The shortened disabilities of the arm, shoulder, and hand questionnaire, VAS: Visual analog scale

	CS	HA	p-value*
Outcome and Time Points	Mean change±SD	Mean change±SD	Between groups
Δ VAS Pain at rest			
Week 6 to baseline	-3.54±1.18	-2.97±1.25	0.001
Week 6 to 12	1.12±1.42	0.51±1.40	0.001
Week 12 to baseline	-2.32±1.10	-2.46±0.97	0.001
Δ Pain at hand grip			
Week 6 to baseline	-4.05±1.18	-3.12±1.88	0.001
Week 6 to 12	0.93±1.23	0.15±1.31	0.001
Week 12 to baseline	-3.12±1.03	-2.97±1.08	0.001
Δ Q-DASH			
Week 6 to baseline	-26.24±11.45	-13.39±10.93	0.001
Week 6 to 12	10.19±12.81	-2.85±10.23	0.014
Week 12 to baseline	-16.05±8.41	-16.24±8.38	0.001
Δ Grip strength			
Week 6 to baseline	19.19±3.65	18.11±4.56	0.001
Week 6 to 12	-4.46±10.79	-3.58±8.6	0.001
Week 12 to baseline	17.58±4.43	15.43±5.3	0.001

Baseline and 12-week MRI scores are represented in Table 4. There were non-significant differences in MRI scores between the two examination points.

**Table 4:** The MRI scores at baseline and 12 weeks MRI: Magnetic resonance imaging, CS: Corticosteroid, HA: Hyaluronate, SD: Standard deviation, *: Student's t-test

MRI Score	CS	HA	p-value*
Baseline (mean±SD)	2.15±0.5	2.05±0.8	0.466
At 12 weeks (mean±SD)	1.80±0.4	1.94±0.6	0.564

## Discussion

This study showed that a single triamcinolone injection was more efficient in short-term pain relief and functional improvements in chronic LE compared to single hyaluronic acid injections. Moreover, a three-month follow-up showed no significant differences between any of the evaluated parameters between treatment groups. The difference in MRI grades was not significantly different in either cohort, confirming that MRI findings are less demonstrative of the disease’s condition in the short term.

The diagnosis of chronic lateral elbow pain is difficult and differential diagnosis had a broad range of clinical conditions such as nerve entrapment, cervical radiculopathy, overuse, and traumatic injuries. A previous systematic review showed high accuracy of several clinical tests such as Cozen’s, Mill’s, and Maudley’s tests and hand grip strength in the diagnosis of LE [14]. However, to decrease selection bias and objectify inclusion criteria, MRI images were also used to support clinical diagnosis in the current study [8]. Primary outcome measures in this study were VAS for pain both at rest and during hand grip strength testing and Q-DASH score. The rationale for using the Q-DASH was that a previous study suggested that the Q-DASH survey was a superior evaluation tool for LE [4]. 

Previous clinical studies on conservative therapy in LE have conflicted results [2]. Corticosteroids were shown to be effective at reducing pain in the short term, with no beneficial effects over a placebo beyond eight weeks [6]. A study comparing steroids with other types of injection materials, i.e., PRP or autologous blood, showed superior results in terms of VAS for pain and functional results in favor of CS in the short term [18]. A systematic review comparing PRP to CS injections confirmed the short-term benefits of CS injections. It also mentioned that studies incorporating CS injection as a treatment had inconsistencies with regard to methodology, used components, or a mixture of components, which might have an effect on the differing clinical results among studies [19]. Another systematic review comparing CS and regenerative injection therapies reported similar improvement in the short term, i.e., one to two months, but better clinical results in favor of the regenerative materials over two years [20]. A systematic review showed that despite the beneficial effects of CS in the short term, they had a negative effect in the intermediate term and conflicting results in the long term. 

Several studies evaluated the effect of HA in the management of LE. One of the leading articles on HA injections in LE stated that after a year of follow-up, peri-articular HA leads to satisfactory outcomes and pain relief compared to placebo. However, VAS for pain decreases in this study were smaller than the minimum clinically important improvement [21]. Contrarily, another study showed a high rate of failure in treatment (25%) when periarticular hyaluronic infiltration was used for resistant cases of lateral epicondylitis [22].

A randomized comparative study on 32 patients with six weeks of follow-up found that dextrose prolotherapy injection was superior to HA in short-term pain relief and functional outcomes [23]. Another randomized placebo-controlled study showed that HA injections were successful in obtaining pain relief by three months and patients continued to have improved outcomes over one year. However, this study was statistically underpowered, excluded saline-injected patients in the analysis due to the high rate of loss to follow-up, and incorporated three HA injections every two weeks [24]. A single HA injection was previously shown to result in pain relief in several enthesopathies, although only 16 patients with LE were included. Hyaluronic acid was shown to be efficient in pain relief, however, the follow-up period was only one week [25]. Another study reported that a mixed injection of HA and CS resulted in better pain relief after six months of follow-up compared to CS alone in a randomized study protocol [14].

The minimal clinically important difference (MCID) for Q-DASH was previously reported as 15.91 [26] and 15.8 for non-operative lateral epicondylitis [27]. The MCID of the VAS for pain was reported as 1.37 and 1.5 [23,28]. The mean clinical improvements were greater than the reported MCID for lateral epicondylitis regarding VAS for pain scores at six and 12 weeks for both triamcinolone and HA groups in the current study. The improvements in Q-DASH score were greater than the MCID in the triamcinolone group both at six and 12 weeks, but only at 12 weeks in the HA group. Besides, differences from six to 12 weeks were less than the MCID for both groups. These data suggest that both triamcinolone and HA had clinically meaningful effects on patients' pain and functional outcome at 12 weeks FU, whereas the HA group showed less improvement in Q-DASH scores at six weeks.

The MRI changes were previously shown to persist at the short-term follow-up in patients injected with PRP and followed up with repeated MRI [17]. Similar to the current study, the authors reported persistent MRI findings despite clinical improvement. Another study showed significantly improved MRI scores on six months FU of patients injected with adipose-derived mesenchymal stromal cells [11]. These findings may reflect that MRI findings are more persistent than the clinical symptoms.

This study has several limitations. There is no placebo injection group and the FU period is short at three months. In line with the study hypothesis, the placebo arm was not planned. It would be interesting to see a placebo-included study with longer follow-up times. Second, the electronic hand dynamometer used in this study is not a validated device, however, all measurements were made using the same device calibrated before every use. The hand grip strength measurements should still be read cautiously when comparisons are made to existing studies, as measurement tools and protocols can cause varying results.

## Conclusions

This study showed that HA injections do not show any clinical efficacy superior to CS, and CS injections relieve pain and improve function more significantly than HA injections in chronic LE in the short term. The HA injections should not be used at an extra cost as no additional beneficiary effects were observed. Injection therapies in chronic LE might be questionable as the treatment effect was short-lived in the current study. Although MRI imaging was useful in the diagnosis, it did not reflect clinical improvements at 12 weeks and it is not recommended to follow up patients with an MRI in the short term.
